# Human biodistribution and radiation dosimetry of the demyelination tracer [^18^F]3F4AP

**DOI:** 10.1007/s00259-022-05980-w

**Published:** 2022-10-05

**Authors:** Pedro Brugarolas, Moses Q. Wilks, Jacqueline Noel, Julia-Ann Kaiser, Danielle R. Vesper, Karla M. Ramos-Torres, Nicolas J. Guehl, Marina T. Macdonald-Soccorso, Yang Sun, Peter A. Rice, Daniel L. Yokell, Ruth Lim, Marc D. Normandin, Georges El Fakhri

**Affiliations:** Department of Radiology, Gordon Center for Medical Imaging, Massachusetts General Hospital, Harvard Medical School, Boston, MA 02114 USA

**Keywords:** [^18^F]3F4AP, 4-Aminopyridine, PET, Biodistribution, Radiation dosimetry, IND, First in human, Demyelination, Multiple sclerosis, Voltage-gated potassium channels, Fluorine-18

## Abstract

**Purpose:**

[^18^F]3F4AP is a novel PET radiotracer that targets voltage-gated potassium (K^+^) channels and has shown promise for imaging demyelinated lesions in animal models of neurological diseases. This study aimed to evaluate the biodistribution, safety, and radiation dosimetry of [^18^F]3F4AP in healthy human volunteers.

**Methods:**

Four healthy volunteers (2 females) underwent a 4-h dynamic PET scan from the cranial vertex to mid-thigh using multiple bed positions after administration of 368 ± 17.9 MBq (9.94 ± 0.48 mCi) of [^18^F]3F4AP. Volumes of interest for relevant organs were manually drawn guided by the CT, and PET images and time-activity curves (TACs) were extracted. Radiation dosimetry was estimated from the integrated TACs using OLINDA software. Safety assessments included measuring vital signs immediately before and after the scan, monitoring for adverse events, and obtaining a comprehensive metabolic panel and electrocardiogram within 30 days before and after the scan.

**Results:**

[^18^F]3F4AP distributed throughout the body with the highest levels of activity in the kidneys, urinary bladder, stomach, liver, spleen, and brain and with low accumulation in muscle and fat. The tracer cleared quickly from circulation and from most organs. The clearance of the tracer was noticeably faster than previously reported in nonhuman primates (NHPs). The average effective dose (ED) across all subjects was 12.1 ± 2.2 μSv/MBq, which is lower than the estimated ED from the NHP studies (21.6 ± 0.6 μSv/MBq) as well as the ED of other fluorine-18 radiotracers such as [^18^F]FDG (~ 20 μSv/MBq). No differences in ED between males and females were observed. No substantial changes in safety assessments or adverse events were recorded.

**Conclusion:**

The biodistribution and radiation dosimetry of [^18^F]3F4AP in humans are reported for the first time. The average total ED across four subjects was lower than most ^18^F-labeled PET tracers. The tracer and study procedures were well tolerated, and no adverse events occurred.

**Supplementary Information:**

The online version contains supplementary material available at 10.1007/s00259-022-05980-w.

## Background

**Fig. 1:**
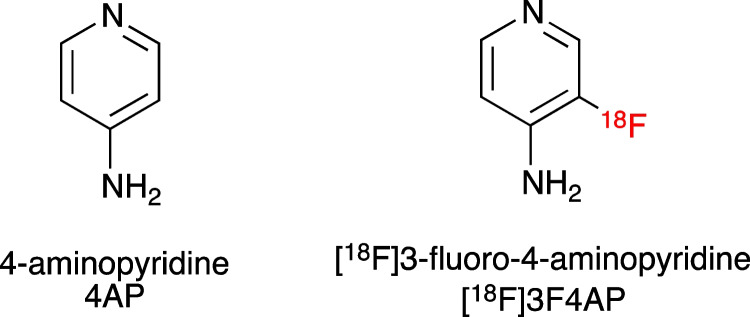
Chemical structures of 4-aminopyridine and [^18^F]3-fluoro-4-aminopyridine

[^18^F]3-fluoro-4-aminopyridine ([^18^F]3F4AP) is a radiofluorinated analog of the multiple sclerosis (MS) drug 4-aminopyridine (dalfampridine, 4AP) [1] (Fig. 1). Similar to 4AP, [^18^F]3F4AP binds to voltage-gated K^+^ channels (K_v_1 family) in demyelinated axons and has been proposed as a PET tracer for imaging demyelinated lesions in the brain. In demyelinated lesions, axonal K^+^ channels K_v_1.1 and K_v_1.2, which are normally buried under the myelin sheath and confined to the juxtaparanodal regions of the axons, become exposed and increase in expression [2–4]. This aberrant expression of K^+^ channels results in excessive efflux of intracellular K^+^ ions and impaired axonal conduction, thus causing neurological deficits in MS and other demyelinating diseases. The FDA-approved drug 4AP binds to and blocks the K^+^ channels in demyelinated axons, reducing the abnormal efflux of K^+^ ions from axons and partially restoring conduction [5–9]. Given the increase in axonal K^+^ channel expression and the ability of 4AP to bind to these channels, [^18^F]3F4AP, a radiofluorinated analog of 4AP, was proposed as a PET tracer for demyelination [1, 10]. [^18^F]3F4AP is similar to 4AP in that it can enter the brain by passive diffusion and bind to K^+^ channels in demyelinated axons. Previous studies in rodents showed that [^18^F]3F4AP can be used to detect lesions in a rat model of demyelination using PET [1]. Additional studies in rhesus macaques showed that [^18^F]3F4AP has suitable properties for imaging primate brains including high brain penetration, fast kinetics, minimal plasma protein binding, and high metabolic stability [11]. Furthermore, PET imaging of a monkey with a small focal traumatic brain injury sustained 3 years prior to imaging showed excellent sensitivity to the lesion [11]. These findings have prompted us to translate [^18^F]3F4AP to human research studies (Clinicaltrials.gov identifiers: NCT04699747, NCT04710550). As the first step in the evaluation of [^18^F]3F4AP in human subjects, we set out to assess the whole-body biodistribution, safety, and radiation dosimetry in healthy human volunteers. We also compare these results with previous findings in nonhuman primates.

## Methods

### Compliance

This study was performed in line with the principles of the Declaration of Helsinki. Approval was granted by the Institutional Review Board at the Massachusetts General Hospital, and the study was registered in clinicaltrials.gov prior to initiation of the study (NCT04710550). [^18^F]3F4AP was administered under an investigational new drug (IND) authorization from the US Food and Drug Administration (FDA).

### Subjects

Four healthy volunteers (2 females, 2 males) participated in the study after providing informed consent. Participating criteria included adults between 18 and 65 years old with no history of brain disease or other serious condition, no contraindications to PET/CT such as severe claustrophobia, accumulated annual radiation dose less than 30 mSv, not taking dalfampridine containing drugs, normal results on a complete metabolic panel (CMP) blood test, normal results on electrocardiogram (ECG), and being able to provide informed consent. No subjects were excluded on the bases of sex, ethnicity, or race.

### Safety assessments

Vital signs including blood pressure, oxygen saturation, and body temperature were collected immediately before and after completion of the scan. Subjects were queried about their level of comfort during and after the scan and were told to report any adverse events on the days following the scan. Within 30 days after completion of the scan, the subjects received a second CMP and a second ECG.

### Radiotracer production

[^18^F]3F4AP was produced by the MGH Gordon PET Core cGMP radiopharmacy using a Neptis ORA synthesizer as recently communicated [12]. The synthesis method is based on the previous report by Basuli et al. [13]. The tracer was purified using a semiprep HPLC column (Waters XBridge C-18, 5 μm, 10 × 250 mm) using 20 mM sodium phosphate (pH 8) mobile phase containing 5% ethanol at a flow rate of 4 mL/min. The HPLC fraction containing the product (approx. 10–11 min) was diluted with 10 mL of 0.9% sodium chloride for injection, USP, and passed through a 0.22 μm sterilizing PES filter into a vented 30 mL sterile empty vial. The product vial was visually inspected, and quality control was performed to FDA and USP standards for chemical identity and purity, radiochemical purity, pH, radionuclidic identity, residual solvents, sterility, and bacterial endotoxins. Identity was confirmed by coinjection of authentic standard on an analytical HPLC column (Phenomenex Gemini C-18, 5 μm, 4.6 × 250 mm) with mobile phase 10 mM sodium phosphate dibasic, 0.25% triethylamine, and 5% acetonitrile at a flow rate of 1 mL/min (*RT* = 10 min). Purity was assessed by area under the curve (AUC) of the product peak at 239 nm relative to other peaks not present in the blank. Molar activity was determined by performing a calibration curve with reference standard using the AUC. The sterile filter used was tested for integrity. The dose was released for injection after passing all quality control tests except for sterility, which was performed after release. All of the batches of [^18^F]3F4AP used in this study met all product specifications, including sterility test.

### Radiotracer administration

The dose was drawn into a syringe, measured and administered intravenously as a 1 min infusion through a catheter in the hand or arm. After administration of the dose, the catheter was flushed with 10 mL of saline and the residual activity in the syringe and catheter measured to calculate the injected dose (ID).

### Image acquisition protocol

Imaging was performed on a GE Discovery MI PET/CT scanner. Subjects were positioned on the bed of the scanner, and a low-dose CT from cranial vertex to mid-thigh was acquired. Based on the CT images, seven bed positions with overlapping edges were selected for PET acquisition (25 cm per bed position with 2.5–5 cm overlap on each end). PET images were acquired over a period of 4 h with two 15-min breaks at approximately 90 min and 160 min after tracer administration. Subjects were encouraged to void their bladder to eliminate radioactive urine during the breaks. After each break, a low-dose CT was performed for anatomical reference and attenuation correction. The PET acquisition protocol consisted of a series of static images at each bed position of increasing duration starting upon administration of the tracer. The full acquisition protocol was as follows: low-dose CT, 4 passes × 1 min PET acquisition per bed position, 4 passes × 2 min/bed, break, 2 passes × 4 min/bed, break, low-dose CT, and 1 pass × 7 min/bed position. After completion of the scan, the PET data was reconstructed using the scanner’s VUE Point HD reconstruction algorithm with 17 subsets and 3 iterations with corrections for scatter, attenuation, deadtime, random coincidence, and scanner normalization.

### Blood sampling and processing

A total of 3–5 mL venous blood samples were taken at approximately 10-, 30-, 60-, 90-, 180-, and 240-min postinjection and kept on ice. About 1 mL of whole blood was transferred to a preweighed tube, weighed, and the radioactivity of whole blood counted using a calibrated gamma counter. Radioactivity per mL (assuming blood density of 1.06 g/mL) was calculated to time of injection (TOI) and normalized to ID. The remaining blood was centrifuged at 1000 g to separate plasma from blood cells. Plasma samples were used for metabolite analysis.

### Radiometabolite analysis

Plasma samples were analyzed using an automated column-switching radio-HPLC system equipped with a radiation detector [14, 15]. Briefly, venous plasma was injected onto the column-switching radio-HPLC and initially trapped on a catch column (Waters Oasis HLB 30 μm) using mobile phase consisting of 99:1 10 mM ammonium bicarbonate pH 8 in water:acetonitrile at 1.8 mL/min. After 4 min, the catch column was backflushed with 96:4 10 mM ammonium bicarbonate pH 8 in water:acetonitrile at 1 mL/min and directed onto a Waters XBridge BEH C-18 (130 Å, 3.5 μm, 4.6 mm × 100 mm) analytical column. Radiochromatograms were exported and plotted using GraphPad.

### Image analysis and dosimetry calculations

The CT and PET images were coregistered to the first CT images. Based on the CT and PET images, volumes of interest (VOIs) were manually drawn using AMIDE software over representative masses of each organ to obtain Bq/cc estimates over the course of the imaging study. The following tissues were included: adrenals, brain, breasts, gall bladder, small intestine, upper and lower large intestine, stomach, heart contents, heart muscle, kidney, liver, lung, muscle, ovaries, pancreas, red marrow, trabecular and cortical bone, spleen, testes, thymus, thyroid, urinary bladder, and uterus. Red marrow was estimated by drawing VOIs within the vertebra in the CT. Whole-body activity was measured with a large VOI covering the entire subject. Subtracting activity measured in the organs listed above yielded an estimate for “remainder in body” in Bq that could be used to compute the residence time. Time-activity curves (TACs) were extracted for each VOI and uncorrected for decay. TACs were extrapolated to ten half-lives after injection by assuming that any further decline in radioactivity occurred only due to physical decay with no biological clearance. Residence times were calculated manually using the extracted Bq/cc values of the PET images and estimates of whole organ masses (from the International Commission on Radiological Protection (ICRP) values) to obtain estimates of Bq/organ. These values were integrated using the trapezoidal method, including integration to ten half-lives past injection. Radiation dosimetry and effective dose were calculated from the integrated TACs using OLINDA software.

## Results

### Participant characteristics and radiotracer injection

The demographics of the volunteers are shown in Table 1. The study included two males and two females between the ages of 20 and 59 years old. Three out of the four subjects self-reported to be white, and one reported more than one race. The body mass index of the participants ranged from 21.2 to 33. Each subject received an intravenous injection of [^18^F]3F4AP (target activity 370 ± 37 MBq) formulated in ~ 10 mL of saline (Table 2). The molar activity of the tracer at the time of injection was 331 ± 122 GBq/μmol (8.93 ± 3.3 Ci/µmol) resulting in injected masses of 3F4AP shown in Table 2. The injected mass was well below the pharmacologically active dose of 4AP (10 mg bid).

**Table 1:** Demographics of study participants

Participant	Sex	Age range (years)	Race/ethnicity	Weight kg (lb)	Height cm (in)	BMI
P1	Female	40–45	Caucasian	79.4 (175)	155 (61)	33
P2	Male	40–45	More than one race	73.5 (162)	175 (69)	24
P3	Male	55–60	Caucasian	61.2 (135)	170 (67)	21.2
P4	Female	20–25	Caucasian	61.2 (135)	163 (64)	23

**Table 2:** Injected activity and effective dose for each participant

Participant	Injected activity MBq (mCi)	Injected mass (microgram)	Effective dose mSv
P1	378.14 (10.22)	0.216	4.20
P2	381.84 (10.32)	0.092	4.89
P3	342.25 (9.25)	0.201	3.29
P4	380.73 (10.29)	0.091	5.60

### Biodistribution and dosimetry

**Fig. 2:**
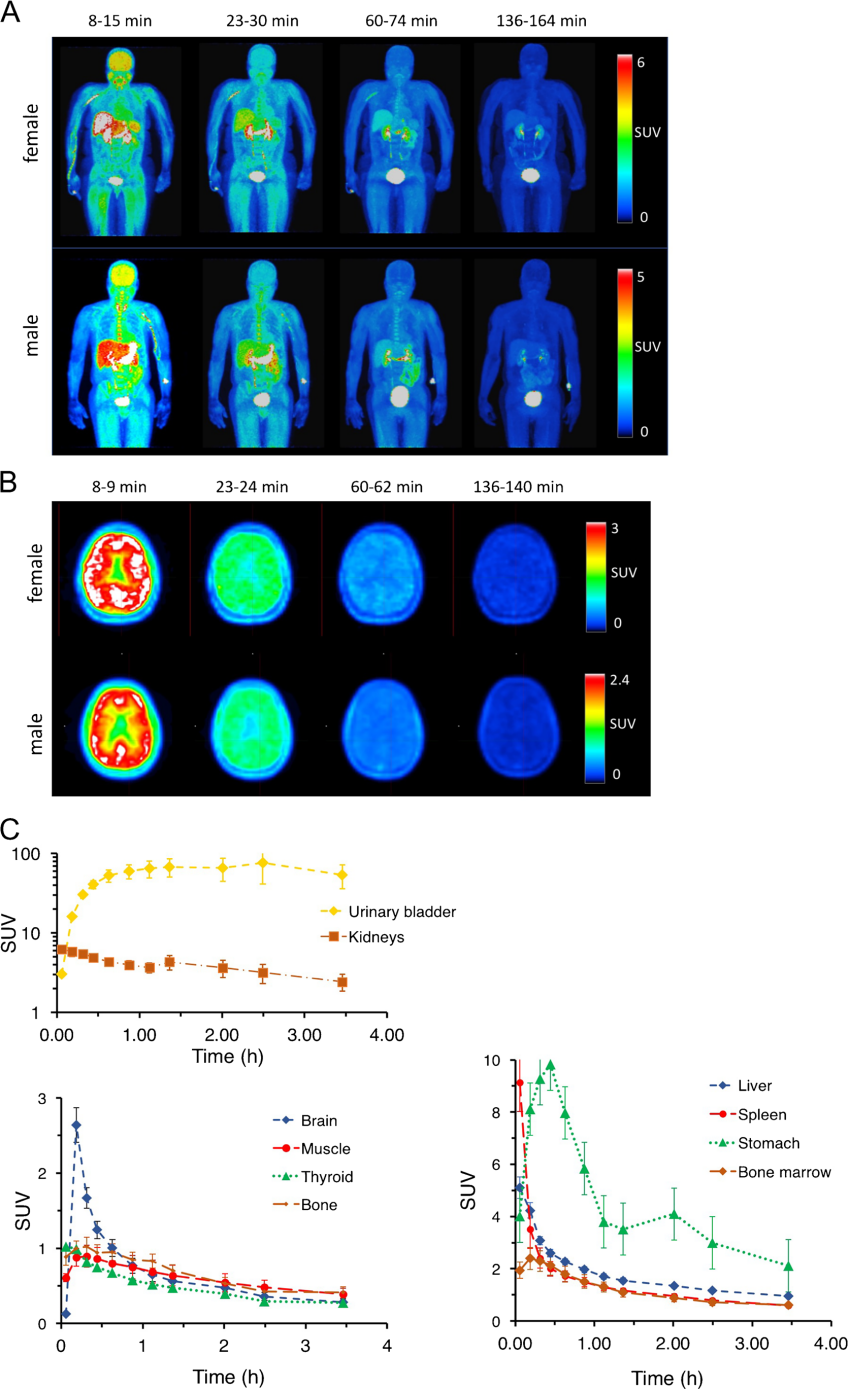
[^18^F]3F4AP in human subjects. **A** Representative whole-body maximum intensity projections of a male and a female participant at different time points. **B** Representative brain images of a male and a female participant. **C** Averaged time-activity curves of selected organs

PET imaging started immediately upon tracer administration and consisted of a series of passes covering the whole body in seven bed positions. To capture the kinetics of the tracer and maximize image quality, the acquisition time per bed position was short at first (1 min per bed position) and increased over time (2, 4, and 8 min per bed position). Whole-body and brain PET images at four selected time points are shown in Fig. 2. As it can be seen from the images and the accompanying time-activity curves (TACs), the tracer is quickly distributed throughout the whole body including the brain. The maximum activity 8–14 min postinjection can be seen in the liver, kidneys, urinary bladder, spleen, stomach, and brain. At 22–28 min post-administration, the highest activity was present in the kidneys, biliary duct, and urinary bladder. After 60 min, most of the activity had cleared from the organs and accumulated in the urinary bladder. Fat was the tissue type with the lowest activity, and no activity accumulation was observed in bone indicative of no defluorination. The biodistribution seen in the whole-body images indicates rapid clearance primarily through the kidneys with a small amount of radioactive tracer or metabolites excreted slowly through the hepatobiliary system. As part of this study, we conducted a preliminary metabolite analysis (Fig. S1) and found substantial metabolism (less than 50% parent remaining 1-h postinjection). This observation was surprising given the lack of metabolism in NHPs (> 90% parent, 3-h postinjection) [11] and the very slow metabolism of 4AP in humans (> 70% parent remaining 24-h post oral administration) [16].

**Fig. 3:**
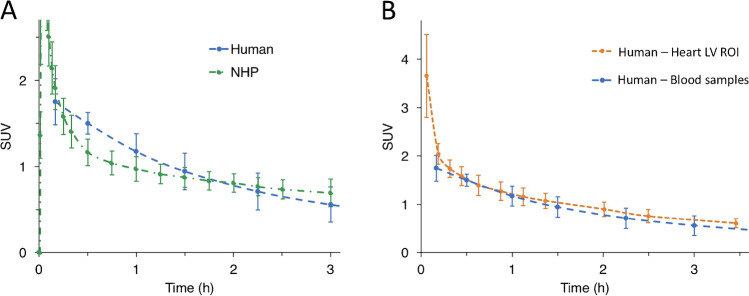
Clearance of [^18^F]3F4AP. **A** Whole blood time-activity curves in human vs. nonhuman primate. **B** Comparison of radioactivity concentration in human blood measured by gamma counting venous samples and the concentration derived from an ROI placed in the heart left ventricle in the PET images

Organ and whole-body dosimetry were calculated from the TACs using OLINDA software as described in the methods (Table 3). Organs with the highest dose were the heart, urinary bladder, and kidneys, whereas the organs with greater contribution to the effective dose were the ovaries, urinary bladder, and testes. The calculated average effective dose was 12.2 ± 2.2 μSv/MBq for the four participants, and no differences were observed between male (11.2 ± 1.6 μSv/MBq) and female (12.9 ± 1.8 μSv/MBq) participants. The effective dose was significantly lower than the effective dose estimated from nonhuman primate studies (21.6 ± 0.6 μSv/MBq) [11] and the effective dose of other PET tracers ([^18^F]FDG ED = ~ 20 μSv/MBq [17]). The lower ED is likely due to the faster clearance in humans than in nonhuman primates. Figure 3A shows averaged whole blood radioactivity concentration curves of the four human subjects and two nonhuman primates and indicates faster clearance in humans. Interestingly, the blood concentration measured from venous blood samples was very similar to the concentration in blood obtained from a VOI placed in the left ventricle of the heart supporting the accuracy of the PET measurements (Fig. 3B).

**Table 3:** Organ dosimetry calculations. Values are mean ± SD of 4 subjects

Target organ	Residence time (h)	Organ dose (μGy/MBq)	Contribution to ED (μSv/MBq)
Adrenals	0.0007 ± 0.0002	14.6 ± 3.6	0.073 ± 0.018
Brain	0.031 ± 0.004	6.7 ± 0.6	0.034 ± 0.003
Breasts	0.004 ± 0.002	4.4 ± 1.1	0.221 ± 0.055
Gallbladder	0.0008 ± 0.0004	8.3 ± 1.0	0
Lower large intestine	0.0058 ± 0.0007	8.9 ± 1.6	1.064 ± 0.196
Small intestine	0.017 ± 0.011	8.3 ± 2.9	0.042 ± 0.015
Stomach	0.024 ± 0.009	14.6 ± 3.6	1.750 ± 0.429
Upper large intestine	0.0052 ± 0.0017	7.2 ± 1.8	0.036 ± 0.009
Heart	0.0106 ± 0.0015	54.6 ± 4.0	0
Kidneys	0.039 ± 0.014	28.6 ± 9.3	0.143 ± 0.046
Liver	0.106 ± 0.018	18.4 ± 1.7	0.922 ± 0.086
Lungs	0.015 ± 0.003	8.4 ± 0.2	1.003 ± 0.027
Muscle	0.52 ± 0.30	7.0 ± 2.3	0.035 ± 0.012
Ovaries	0.0007 ± 0.0005	14.9 ± 4.5	2.965 ± 0.898
Pancreas	0.0044 ± 0.0006	15.5 ± 2.3	0.077 ± 0.011
Red marrow	0.052 ± 0.020	8.6 ± 2.1	1.034 ± 0.248
Trabecular bone	0.015 ± 0.004	13.2 ± 1.6	0.132 ± 0.016
Cortical bone	0.086 ± 0.024	2.6 ± 0.6	0.026 ± 0.006
Spleen	0.009 ± 0.002	15.0 ± 3.4	0.075 ± 0.017
Testes	0.0015 ± 0.004	9.8 ± 2.2	1.950 ± 0.438
Thymus	0.0006 ± 0.0001	11.7 ± 1.6	0.059 ± 0.008
Thyroid	0.0003 ± 0.0003	5.4 ± 2.2	0.271 ± 0.110
Urinary bladder	0.0833 ± 0.0426	51.4 ± 30.3	2.568 ± 1.512
Uterus	0.0015 ± 0.0015	9.4 ± 6.9	0.047 ± 0.034
		Total effective dose	12.1 ± 2.2 μSv/MBq

### Safety assessment

No significant changes in vital signs (temperature, blood pressure, and oxygen saturation) were observed before and after the scan (Table S1). In addition, no significant changes were noted in blood CMP results and electrocardiogram results obtained within 30 days before and 30 days after (Tables S2–S5). The tracer and the imaging procedure were well tolerated by all the participants, and no adverse events occurred during the scan.

## Discussion

We present results from the first study with [^18^F]3F4AP in human research subjects. The goal of this study was to assess the biodistribution, radiation dosimetry, and safety of [^18^F]3F4AP in healthy volunteers. The study included four healthy adults (two males and two females). The biodistribution and calculated effective doses across subjects were found to be very consistent justifying scanning a small number of participants. The tracer was found to distribute widely throughout the body including into the brain and clear quickly primarily via renal excretion. The clearance of the tracer was faster than expected from a previous study in rhesus macaques as evidenced by a faster drop in blood concentration compared to NHPs. The faster clearance may be due to metabolism of the tracer as observed on our preliminary metabolism analysis. Based on prior studies in primates showing > 90% parent 3-h postinjection metabolism, we did not expect significant metabolism; however, there could be species differences in the metabolism of [^18^F]3F4AP, and NHP studies were performed under isoflurane anesthesia, which could partially inhibit the metabolism of the tracer. The faster metabolism and clearance of [^18^F]3F4AP are worth investigating further. This faster clearance likely resulted in lower organ doses and a lower effective dose than predicted from NHP studies.

Although this study only included a small number of volunteers, the tracer and study procedures were well tolerated among study participants as it is expected for PET tracers. In addition, this study showed that the radiation dose in humans is within the acceptable levels, and that the tracer readily enters the brain opening the door for additional studies investigating the ability of [^18^F]3F4AP to detect demyelinated lesions in different patient populations. Currently, clinicaltrials.gov lists two studies focused on evaluating [^18^F]3F4AP in patients with multiple sclerosis (NCT04699747) as well as Alzheimer’s disease, mild cognitive impairment, and traumatic brain injury (NCT04710550). Given that demyelination occurs in these and many other diseases and it is potentially reversible, a tracer that allows quantitative imaging of demyelination in humans may contribute to better understanding of the pathophysiological processes, more accurate diagnoses of brain diseases, and provide a tool for accurately monitoring remyelinating therapies.

## Supplementary Information

Below is the link to the electronic supplementary material.

Supplementary file1 (DOCX 1312 KB)

## Data Availability

The datasets generated during and analyzed during the current study are available from the corresponding author on reasonable request.
