# Supplementary material for: Human biodistribution and radiation dosimetry of the demyelination tracer [18F]3F4AP
Source: Eur J Nucl Med Mol Imaging. 2022 Oct 5;50(2):344–51. doi: 10.1007/s00259-022-05980-w (PMC9816249; doi:10.1007/s00259-022-05980-w)

SUPPLEMENTAL INFORMATION

**Human biodistribution and radiation dosimetry of the demyelination tracer [^18^F]3F4AP**

Pedro Brugarolas^#,*^, Moses Q. Wilks^#^, Jacqueline Noel, Julia-Ann Kaiser, Danielle R. Vesper, Karla M. Ramos-Torres, Nicolas J. Guehl, Marina T. Macdonald-Soccorso, Yang Sun, Peter A. Rice, Daniel L. Yokell, Ruth Lim, Marc D. Normandin^¶^ and Georges El Fakhri^¶,*^

Gordon Center for Medical Imaging, Department of Radiology, Massachusetts General Hospital and Harvard Medical School, Boston, MA, USA.

Pedro Brugarolas, Ph.D.

Gordon Center for Medical Imaging

Massachusetts General Hospital

Harvard Medical School

55 Fruit Street, Bulfinch 051

Boston, MA 02114

E-mail: [pbrugarolas@mgh.harvard.edu](mailto:pbrugarolas@mgh.harvard.edu)

Georges El Fakhri, Ph.D., DABR

Gordon Center for Medical Imaging

Massachusetts General Hospital

Harvard Medical School

125 Nashua Street, Suite 660

Boston, MA 02114

E-mail: [elfakhri.georges@mgh.harvard.edu](mailto:elfakhri.georges@mgh.harvard.edu)

**SUPPLEMENTARY MATERIALS**

| **Item** | **Description** | **Pages** |
| --- | --- | --- |
| Table S1 | Vital signs pre- and post- scan for the participants | 3 |
| Tables S2-S5 | CMP results before and after the scan | 4, 5 |
| Fig. S1 | Representative radiochromatogram of venous plasma samples. | 6 |

**Table S1. Vital signs pre and post scan for the participants.**

| **Subject P1** | | | |
| --- | --- | --- | --- |
| **Pre-Injection Vitals** | | **Post Scan Vitals** | |
| Blood Pressure | 117/79 | Blood Pressure | 117/80 |
| Heart Rate (BPM) | 70 | Heart Rate (BPM) | 69 |
| SpO2 (%) | 99 | SpO2 (%) | 98 |
| Respiration Rate (BPM) | 20 | Respiration Rate (BPM) | 16 |
| Temperature (°F) | 96.3 | Temperature (°F) | 97.7 |
|  |  |  |  |
| **Subject P2** | | | |
| **Pre-Injection Vitals** | | **Post Scan Vitals** | |
| Blood Pressure | 136/96 | Blood Pressure | 144/98 |
| Heart Rate (BPM) | 68 | Heart Rate (BPM) | 72 |
| SpO2 (%) | 96 | SpO2 (%) | 100 |
| Respiration Rate (BPM) | 16 | Respiration Rate (BPM) | 16 |
| Temperature (°F) | 97.3 | Temperature (°F) | 97.9 |
|  |  |  |  |
| **Subject P3** | | | |
| **Pre-Injection Vitals** | | **Post Scan Vitals** | |
| Blood Pressure | 126/80 | Blood Pressure | 123/83 |
| Heart Rate (BPM) | 91 | Heart Rate (BPM) | 85 |
| SpO2 (%) | 99 | SpO2 (%) | 97 |
| Respiration Rate (BPM) | 12 | Respiration Rate (BPM) | 13 |
| Temperature (°F) | 97.2 | Temperature (°F) | 98.4 |
|  |  |  |  |
| **Subject P4** | | | |
| **Pre-Injection Vitals** | | **Post Scan Vitals** | |
| Blood Pressure | 107/71 | Blood Pressure | 104/69 |
| Heart Rate (BPM) | 82 | Heart Rate (BPM) | 66 |
| SpO2 (%) | 99 | SpO2 (%) | 100 |
| Respiration Rate (BPM) | 17 | Respiration Rate (BPM) | 12 |
| Temperature (°F) | 98.6 | Temperature (°F) | 98.3 |

**Tables S2-S5. CMP results before and after the scan**

Subject P1 (CMP obtained 17 days before and 6 days after the scan)

|  |
| --- |
| 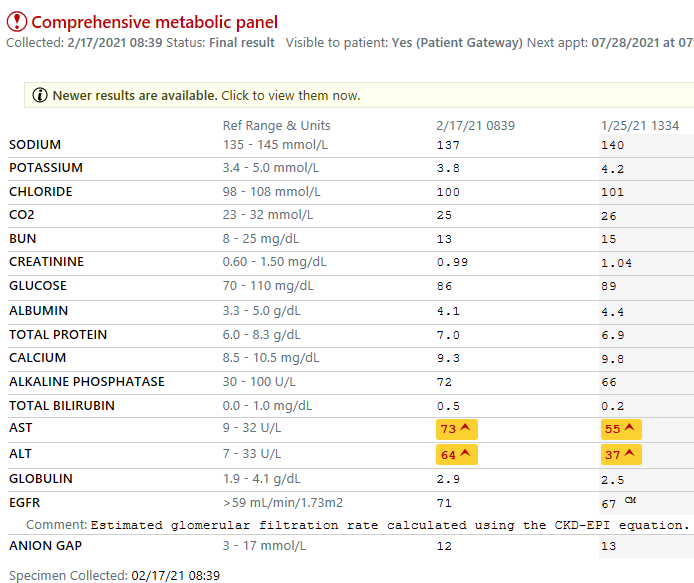 |
|  |
| Subject P2 (CMP obtained 21 days before and 6 days after the scan) |
| 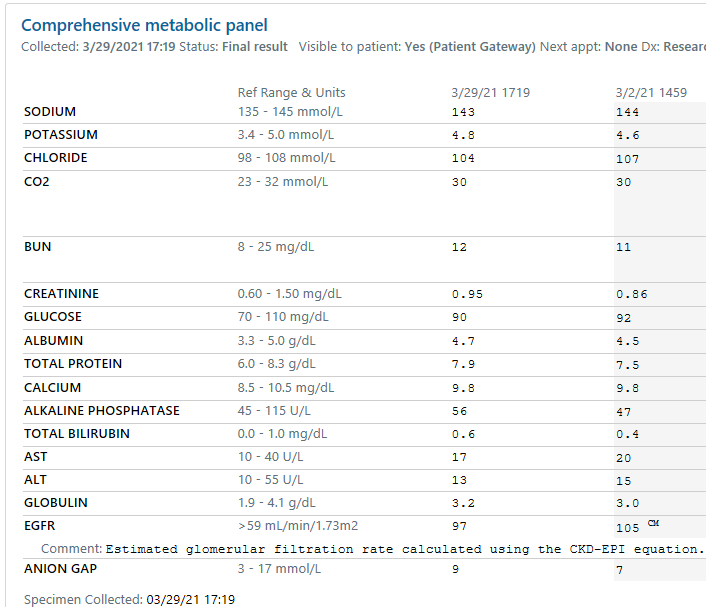 |
|  |
| Subject P3 (CMP obtained 36 days before and 31 days after the scan)  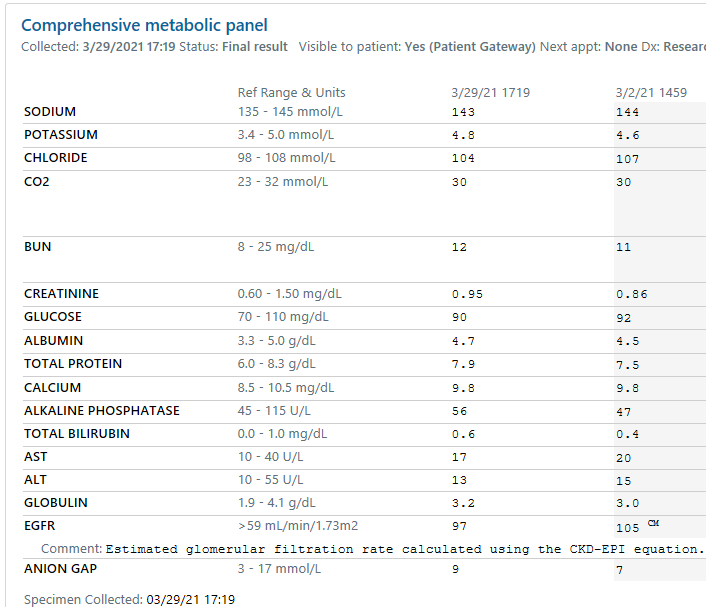  Subject P4 (CMP obtained 5 days before and 2 days after the scan) |
|  |
| 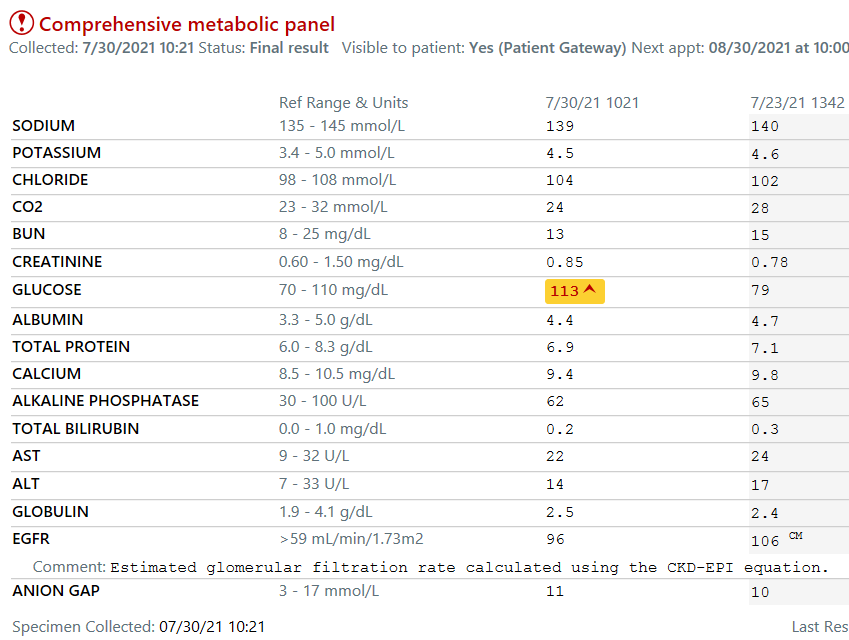 |
|  |

**Fig S1**. Representative radiochromatogram of venous plasma samples.


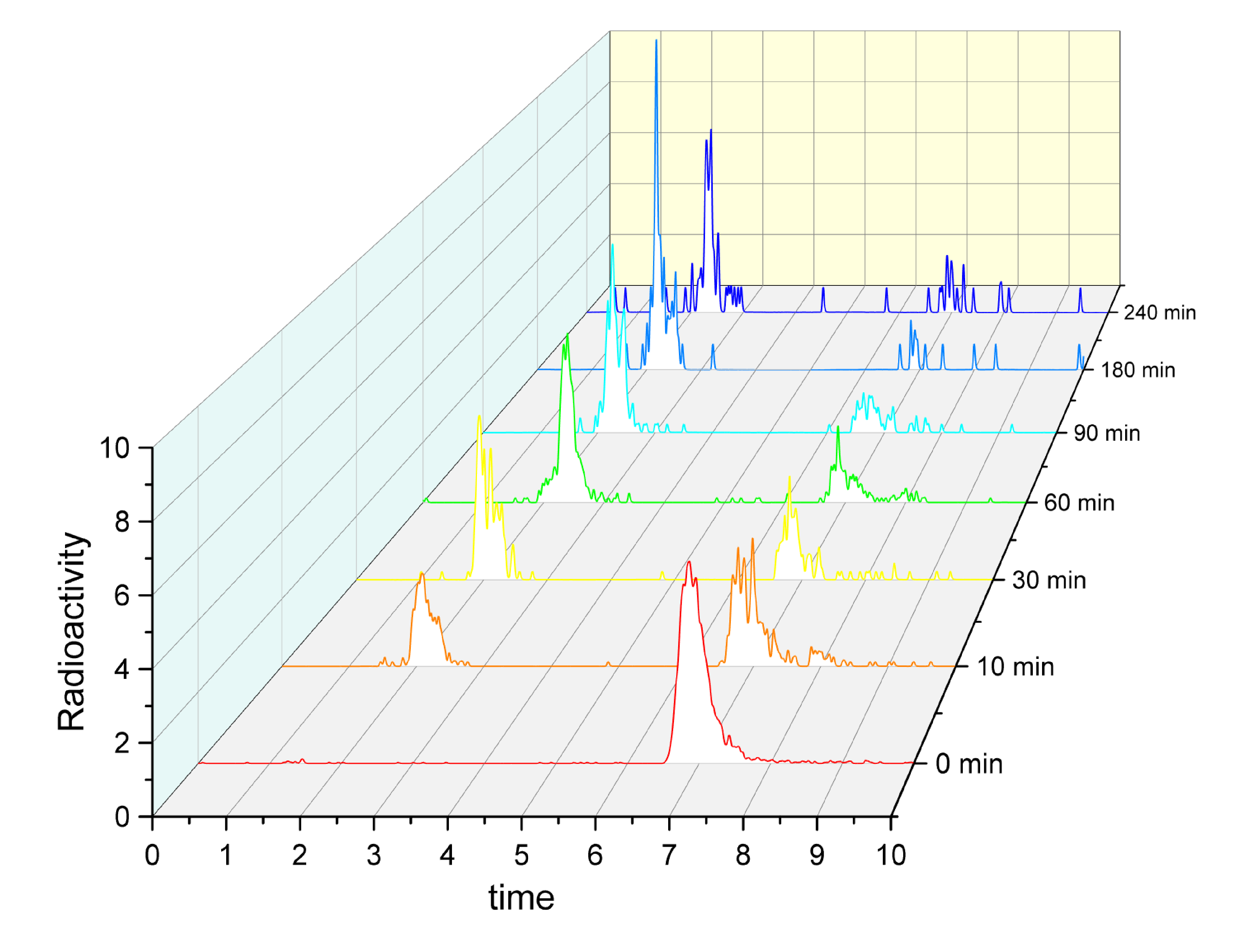

Supplement: Supplementary file 1 — Supplementary file1 (DOCX 1312 KB) [file 259_2022_5980_MOESM1_ESM.docx]
